# Whole genome sequencing of a clinical drug resistant *Candida albicans* isolate reveals known and novel mutations in genes involved in resistance acquisition mechanisms

**DOI:** 10.1099/jmm.0.001351

**Published:** 2021-04-28

**Authors:** Roy A. Khalaf, Nour Fattouh, Matej Medvecky, Jaroslav Hrabak

**Affiliations:** ^1^​ Department of Natural Sciences, Lebanese American University, PO Box 36, Byblos, Lebanon; ^2^​ Institute of Biodiversity, Animal Health and Comparative Medicine, University of Glasgow, Glasgow, UK; ^3^​ Department of Microbiology, Faculty of Medicine, University Hospital in Pilsen, Charles University, 32300 Pilsen, Czech Republic; ^4^​ Biomedical Center, Faculty of Medicine in Pilsen, Charles University, 32300 Pilsen, Czech Republic

**Keywords:** antifungal resistance, azoles, *Candida albicans*, ergosterol, virulence, whole genome sequencing

## Abstract

*Candida albicans* is an opportunistic pathogen accounting for the majority of cases of *Candida* infections. Currently, *C. albicans* are developing resistance towards different classes of antifungal drugs and this has become a global health burden that does not spare Lebanon. This study aims at determining point mutations in genes known to be involved in resistance acquisition and correlating resistance to virulence and ergosterol content in the azole resistant *C. albicans* isolate CA77 from Lebanon. This pilot study is the first of its kind to be implemented in Lebanon. We carried out whole genome sequencing of the azole resistant *C. albicans* isolate CA77 and examined 18 genes involved in antifungal resistance. To correlate genotype to phenotype, we evaluated the virulence potential of this isolate by injecting it into BALB/c mice and we quantified membrane ergosterol. Whole genome sequencing revealed that eight out of 18 genes involved in antifungal resistance were mutated in previously reported and novel residues. These genotypic changes were associated with an increase in ergosterol content but no discrepancy in virulence potential was observed between our isolate and the susceptible *C. albicans* control strain SC5314. This suggests that antifungal resistance and virulence potential in this antifungal resistant isolate are not correlated and that resistance is a result of an increase in membrane ergosterol content and the occurrence of point mutations in genes involved in the ergosterol biosynthesis pathway.

## Data Summary

The authors confirm that all protocols have been provided within the article and relevant supplementary material deposited in GenBank. Deposition and accession numbers are also provided.

## Introduction

Candida is a genus of fungi that is part of the normal human microbiota, colonizing many bodily parts such as the gut and skin [1]. In immunocompromised hosts, *Candida* is an opportunistic pathogen [2], leading to deadly disseminated systemic infections in its most severe form [3]. In hospitalized patients, the diploid *Candida albicans* species causes the majority of cases of *Candida* infections [1].

Generally, *C. albicans* infections are treated with topical or systemic antifungal drugs belonging to the following classes: allylamines, azoles, echinocandins, morpholines, and polyenes [4]. Allylamines, azoles, and morpholines interfere with genes that code for enzymes having crucial roles within the ergosterol biosynthesis pathway. This yeast metabolic pathway allows the conversion of acetyl coenzyme A into the lipid ergosterol which is the main component of the *C. albicans* cell membrane. Within the ergosterol biosynthesis pathway, allylamines and azoles mainly target the *ERG1* and *ERG11* genes, respectively while morpholines mainly target the *ERG24* and *ERG2* genes [5, 6]. Regarding polyenes, they act by directly binding to ergosterol at the level of the *C. albicans* cell wall rendering it more permeable. Echinocandins, on the other hand, interfere with the cell wall component glucan since they mainly inhibit the action of beta-1,3-D-glucan synthase [7]. In fact, resistance towards antifungal drugs relies on many mechanisms. The two main mechanisms involved are: (1) Alterations at the level of target genes such as the genes that code for squalene epoxidase and lanosterol 14-alpha demethylase among others. This alteration in target genes is the outcome of an overexpression or point mutations. Overexpression of target genes allows to compensate for the inhibition of its protein product caused by the antifungal drug while point mutations lead to a reduced affinity of antifungal drugs to the target protein. (2) Changes in cell wall and plasma membrane permeability due to changes in their composition, an active efflux or a reduced import of antifungals. In addition to the two main mechanisms mentioned above; metabolic bypass, regulation of oxidative stress response, regulation of thermal stress, and metal deficiency could be at the basis of antifungal resistance mechanisms in *C. albicans* [8]. The development of resistance towards antifungals in *C. albicans* is on the rise as a result of an improper management and treatment of infections and the challenging task of discovering new antifungals since *C. albicans* have relatively high rates of gene orthology with humans [9, 10]. Consequently, *C. albicans* infections pose a serious public health threat worldwide including Lebanon. To date only a handful of studies have addressed resistance of Lebanese *C. albicans* isolates to azoles, echinocandins, and polyenes [11–17] but none of the studies have looked at the entire genome of *C. albicans* to generate a complete picture of the genomic aspects of antifungal resistance.

**Fig. 1.**
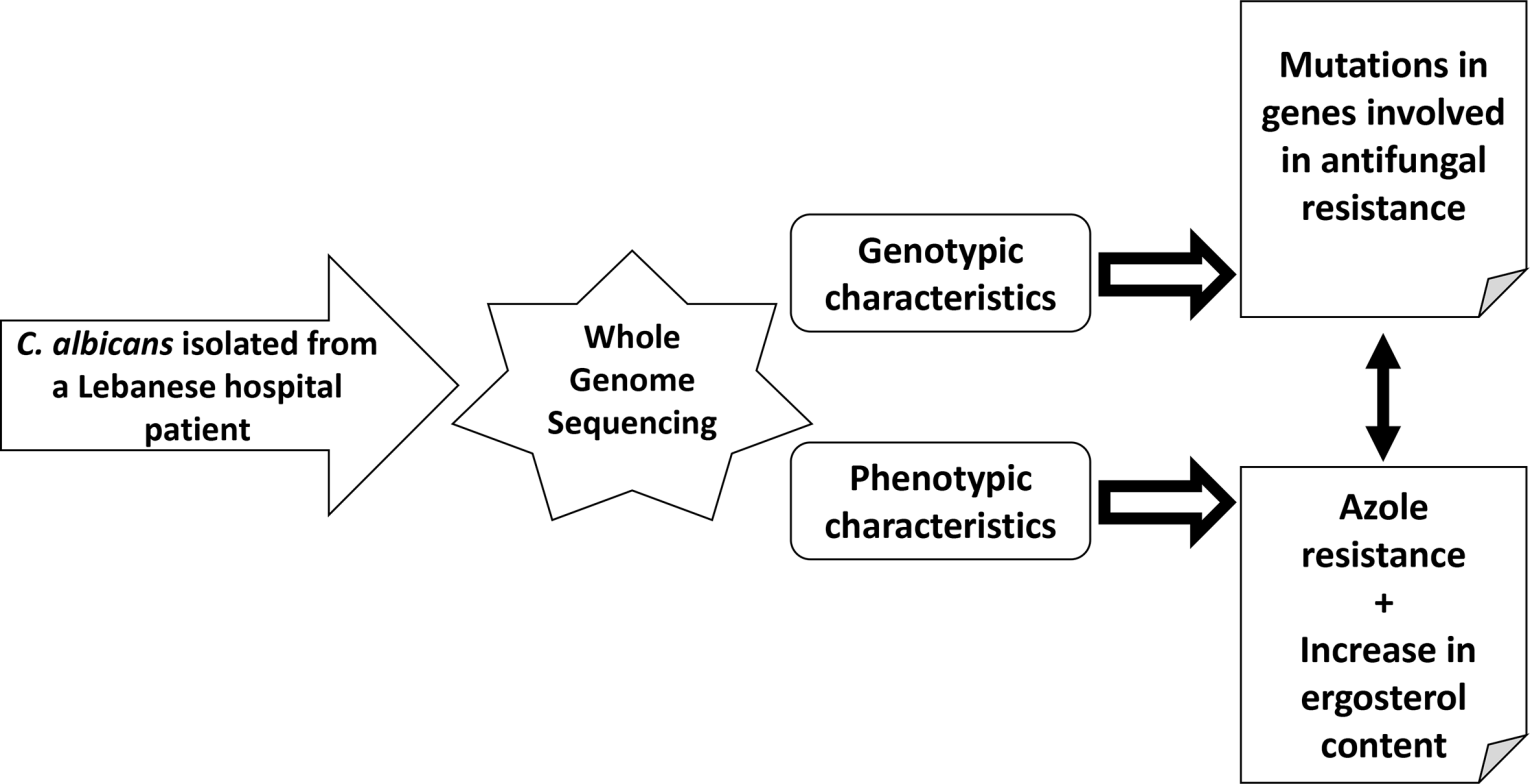
Summary. The *C. albicans* resistant strain was whole genome sequenced, and membrane ergosterol levels determined. Increase in ergosterol content partially explains the increased drug resistance phenotypes observed. Point mutations in key resistance genes were also isolated.

The aim of this pilot study was to perform whole genome sequencing of an antifungal resistant *C. albicans* clinical isolate from Lebanon in order to analyse a significant number of genes involved in antifungal resistance at once. We focused on 18 genes of which eight (*ERG1*, *ERG11*, *ERG24*, *ERG251*, *ERG6*, *ERG2*, *ERG3*, *ERG5*) code for crucial proteins within the ergosterol biosynthesis pathway [5, 6, 18], one (*UPC2*) codes for a regulator of many genes involved in the ergosterol biosynthesis pathway, three (*CDR1*, *CDR2*, and *MDR1*) code for efflux pumps, three (*TAC1*, *MRR1*, and *MRR2*) code for efflux pump regulators [8, 19], two (*FKS1* and *FKS2*) code for different subunits of beta-1,3-D-glucan synthase which is involved in the assembly of the beta-1,3-glucan at the level of the cell wall (7), and one (*GLS1*) codes for alpha-glucosidase I which is implicated in the assembly of the beta-1,6-glucan cell wall component [20]. Most of these targets are considered to be cell surface proteins [21]. In parallel, the genotypic data generated for this resistant *C. albicans* isolate were correlated to the following phenotypic aspects: minimum inhibitory concentration (MIC) of some azoles (fluconazole, itraconazole, ketoconazole, and voriconazole), MIC of the echinocandin caspofungin, MIC of the polyene amphotericin B, virulence potential, and membrane ergosterol content. Fig. 1 summarizes the methodology and results of this pilot study.

## Methods

### Case presentation

The *C. albicans* strain (designated as CA77) was isolated from a stool sample of a patient admitted in a tertiary care center in Beirut, Lebanon. The patient was suffering from diarrhea and did not have any history of antibiotic treatment before being admitted.

### Antifungal resistance

Susceptibility to fluconazole (FL), itraconazole (IT), ketoconazole (KE), voriconazole (VO), amphotericin B (AP), and caspofungin (CS) was evaluated for CA77 using the E-test (BioMérieux) method according to the manufacturer’s instructions. *Candida parapsilosis* ATCC 22019 and *Candida albicans* ATCC 90028 were used as quality control standards with our values falling within the CLSI approved range [22].

### Virulence potential

CA77 was inoculated onto potato dextrose agar (Conda Laboratories, Madrid, Spain) and incubated for 48h at 30 °C. Then, 10 ml of potato dextrose broth (Conda Laboratories, Madrid, Spain) was inoculated with one colony of CA77 and incubated for 18h at 30 °C with shaking at 100 r.p.m. Then 10^7^ cells were harvested and suspended in 0.2 ml of a 1× phosphate-buffered saline solution and injected into the tail vein of 6-week-old female BALB/c mice in order to induce a disseminated systemic infection. Water and food were administered *ad libitum*. In total, six BALB/c mice were injected with CA77. The mice were monitored on a daily basis for a period of 1 month and the number of moribund mice was counted. Moribund mice were euthanized [15, 23–25]. The same protocol was carried out for the reference *C. albicans* SC5314 strain (ATCC MYA-2876) known to be susceptible to all antifungals.

### Quantification of membrane ergosterol

CA77 was inoculated onto potato dextrose agar (Conda Laboratories, Madrid, Spain) and incubated for 48h at 30 °C. Then, 50 ml of potato dextrose broth (Conda Laboratories, Madrid, Spain) was inoculated with one colony of CA77 and incubated for 17h at 35 °C with shaking at 100 r.p.m. The extraction and quantification of ergosterol from CA77 followed the protocol described in [26]. Briefly, yeast cells were collected by centrifugation at 2700 r.p.m. for 5 min and washed once with sterile distilled water. The wet weight of the yeast pellet was measured. A saponification reaction was carried out by adding 3 ml of a 25% alcoholic potassium hydroxide solution to the weighed pellet of yeast cells. The suspension was vortexed for 1 min, transferred to a glass screw-cap tube, and incubated in a water bath for 1h at 85 °C. The tube was cooled down at room temperature for 15 min. The nonsaponifiable sterols were extracted by adding 1 ml of sterile distilled water and 3 ml of *n*-heptane. The mixture was vortexed for 3 min and incubated at room temperature for 1h to allow phase separation. The *n*-heptane layer was transferred to a clean glass screw-cap tube and incubated at -20 °C for 24h. A fivefold dilution of the *n*-heptane layer in 100% ethanol was performed and optical density measurements at 230 and 281.5 nm were taken using a Genesys 10S UV-Vis spectrophotometer. The equations in [26] were used to calculate the ergosterol content as a percentage of the wet weight of yeast cells. The same protocol was carried out for the reference *C. albicans* SC5314 strain (ATCC MYA-2876) that is known to be susceptible to all antifungals. The experiment was performed in biological triplicates and the average percentage ergosterol content was calculated for each isolate. The percentage change in ergosterol in the CA77 strains in comparison to the SC5314 strain was calculated.

### DNA extraction

Whole genome DNA was extracted using the NucleoSpin Microbial DNA kit (Macherey-Nagel, Duren, Germany). The obtained DNA was sheared using the Covaris g-TUBEs (Covaris, USA). Library preparation was performed on the sheared DNA in accordance with the manufacturer’s recommendation for microbial multiplexing protocol (Pacific Biosciences, Menlo Park, CA, USA). No size selection was performed during the library preparation. The constructed library was sequenced using long-read sequencing technology on Sequel I (Pacific Biosciences, Menlo Park, CA, USA).

### Whole genome sequence data analysis

Initial PacBio long reads were pre-assembled and error corrected using HGAP4 offered through SMRT Link v.6.0 [27]. Pre-assembled reads were then assembled using FALCON (falcon-kit v1.8.0) with configuration files optimized for the purposes of yeast genome assembly using default settings. Finally, haplotype phasing was extended via FALCON-Unzip v1.3.5 using default settings [28]. Assemblies and/or reads were mapped against *C. albicans* SC5314 (GenBank accession numbers: NC_032089-NC_032096) using blast and Bowtie 2 v2.3.4.2 [29, 30] to investigate the completeness of the assembly across the different chromosomes. Comparison of our sequence with that of the Candida Genome Database to detect single nucleotide polymorphisms was done using Mauve [31].

## Results

In this pilot study, one *C. albicans* clinical isolate referred to as CA77 was screened for its capacity to resist the common antifungal azole drugs FL, IT, KE, and VO, in addition to the polyene AP, and the echinocandin CS by relying on the E-test method. We observed that CA77 was resistant to all tested azole antifungals but was susceptible to AP and CS. The MICs for all tested drugs are listed in Table 1.

**Table 1.** Susceptibility of the *C. albicans* clinical isolate CA77 to common antifungal drugs. Minimum inhibitory concentration (MICs) obtained via the E-test method for fluconazole (FL), itraconazole (IT), ketoconazole (KE), and voriconazole (VO) show that the CA77 isolate is resistant to those antifungal drugs while MICs for amphotericin B (AP) and caspofungin (CS) show that it is susceptible to those drugs

MICs (μg ml^−1^)					
**FL**	**IT**	**KE**	**VO**	**AP**	**CS**
>256	>32	>32	>32	0.125	0.25

Whole genome sequencing partially phased assembly resulted in a genome size of 14 133 179 bp with a GC content of 33.67%. When aligned against the reference genome SC5314, 95.7% coverage query was observed. Around 38 000 single nucleotide polymorphisms were detected throughout the genome of the CA77 isolate, accounting for 0.27% of its genome. Previous studies found that single nucleotide polymorphisms account for 0.4 to 0.8% of the genome compared to the reference *C. albicans* strain SC5314 [32]. To generate a complete picture of antifungal resistance mechanisms at play in CA77 from a genetic perspective, 18 genes involved in antifungal resistance (*ERG1*, *ERG11*, *ERG24*, *ERG251*, *ERG6*, *ERG2*, *ERG3*, *ERG5*, *UPC2*, *CDR1*, *CDR2*, *MDR1*, *TAC1*, *MRR1*, *MRR2*, *FKS1*, *FKS2*, and *GLS1*) were investigated. Whole genome sequencing showed that the following eight genes harbored point mutations: *ERG11*, *ERG24*, *ERG251*, *UPC2*, *CDR1*, *MRR2*, *FKS1*, and *GLS1*. Some of these genes exhibited more than one point mutation and all mutations were homozygous with no heterozygosity observed. All mutations are listed in Table 2. In total, 19 point mutations were observed of which to our knowledge, only nine were previously reported but do not have any clear implication in antifungal resistance.

**Table 2.** A list of known and novel mutations detected by whole genome sequencing in genes involved in antifungal resistance in the *C. albicans* clinical isolate CA77

Genes	Mutations	Mutation description	References
*ERG11*	D116E	Known mutation but not previously involved in antifungal resistance	[36]
*ERG24*	H46Q – I88V – A307V	Novel mutations	–
*ERG251*	R310Q	Novel mutation	–
*UPC2*	I142S	Known mutation but not previously involved in antifungal resistance	[36]
*CDR1*	T842S – T950S – E948P – I916V	Known mutations but not previously involved in antifungal resistance	[37]
*MRR2*	V451A – S480P	Known mutations but not previously involved in antifungal resistance	[38]
*FKS1*	P1838I	Novel mutation	–
	T1886S	Known mutation but not previously involved in antifungal resistance	[36]
*GLS1*	T181I – I340V – Q459R – M679I – T938M	Novel mutations	–

Since most of these mutations are at the level of genes involved in the ergosterol biosynthesis pathway, we then decided to determine whether an association exists between the mutations and an increase in membrane ergosterol content. We observed a 27% increase in membrane ergosterol content compared to the control SC5314 strain and this change in membrane composition could likely lead to impermeability towards azoles. Table 3 shows the ergosterol content in percentages in both CA77 and SC5314 *C. albicans* strains. Regarding virulence potential, no difference was observed between our resistant CA77 isolate and the control reference strain SC5314 that is susceptible to all antifungals since in both cases four out of six BALB/c mice were moribund following a disseminated systemic infection.

**Table 3.** Ergosterol content in *C. albicans* clinical isolate CA77 and reference strain SC5314. The average ergosterol content of the three biological triplicates along with the standard deviation are listed in this table

*C. albicans* strain	Average ergosterol content in %	Standard deviation
**SC5314**	0.0058	0.0017
**CA77**	0.0074	0.0013

## Discussion

Whole genome sequencing has been used intensively in sequencing microbes especially in prokaryotes where data analysis is straightforward. However, diploid organisms pose challenges especially in extracting heterozygous loci [33]. In this study, heterozygosity in 18 genes has been investigated. We conducted a whole genome sequencing approach using long-reads sequencing; the first of its kind in Lebanon, to achieve a better assembly covering longer segments of the chromosomes and to determine the molecular basis of drug resistance in a Lebanese hospital isolate. The isolate exhibited resistance to the most commonly used azoles such as FL, IT, KE, and VO. We observed point mutations in eight genes involved in antifungal resistance: *ERG11*, *ERG24*, *ERG251*, *UPC2*, *CDR1*, *MRR2*, *FKS1*, and *GLS1*. In total, 19 point mutations were detected, of which nine were known to be uninvolved in antifungal resistance and ten were novel. Mutations in genes involved in the ergosterol biosynthetic pathway might change the 3D structure of the enzyme preventing azoles from binding, resulting in resistance. The novel mutations were detected in *ERG24*, *ERG251*, *FKS1*, and *GLS1*. Our E-test results show that the CA77 isolate is resistant to FL, IT, KE, and VO that belong to the azole class even though all four genes experiencing novel mutations are not azole targets. Nonetheless, the development of resistance towards azoles could be explained by ergosterol content that shows a 27% increase compared to the reference strain SC5314. This leads to a change in the membrane composition of CA77 likely rendering it impermeable to azole antifungals that were no longer able to reach their target within the *C. albicans* cell. However, other studies did not detect an increase in ergosterol content in azole-resistant compared to azole-susceptible *C. albicans* isolated from humans and animals [34, 35]. In addition to azole resistance, we speculate that this strain might be resistant to morpholine antifungals that could have *ERG24* as target [5, 6]. The *ERG251* gene is not a direct antifungal target [18] so, it is difficult to speculate about the implication of mutations at the level of this gene on antifungal resistance. Mutations at the level of *ERG24* and *ERG251* are rarely observed. Moreover, *FKS1* and *GLS1* are genes that code for a subunit of beta-1,3-D-glucan synthase and alpha-glucosidase I involved in the assembly of the cell wall beta-1,3-glucan and beta-1,6-glucan, respectively. In general, point mutations at the level of those genes could reduce sensitivity to echinocandins [7, 20]. However, we observed no correlation between antifungal resistance and the virulence potential of CA77 in contrast to what has been observed in *C. albicans* clinical isolates from Lebanon that were resistant to CS. These isolates exhibited a lower virulence potential which was associated with an increased chitin deposition at the level of the cell wall rendering it thicker and thus, preventing filamentation which is crucial for the establishment of virulence. However, most ergosterol enzymes, as opposed to enzymes targeted by echinocandins, are not associated with the cell wall so such a correlation is not to be expected [15]. In light of an absence of change in virulence potential between CA77 and the reference strain SC5314 that is susceptible to all antifungal drugs we could postulate that the novel mutations harbored by *FKS1* and *GLS1* are probably not involved in resistance to echinocandins and definitely not implicated in resistance to the echinocandin CS according to our E-test results.

## Conclusion

In conclusion, the *C. albicans* CA77 strain isolated from a hospitalized patient in Lebanon and which is resistant to the most commonly used azole antifungals was subjected to a whole genome sequencing analysis which revealed the occurrence of 19 point mutations in eight genes involved in antifungal resistance. Of these mutations, nine were previously documented and known to be uninvolved in the development of antifungal resistance and ten were novel mutations but not harbored by azole targets. This development of resistance towards azoles is thought to be the result of a change in membrane composition due to an increase in ergosterol content (Fig. 1). Finally, our whole genome sequencing pilot study is the first of its kind to be carried out in Lebanon and will pave the way to a large-scale project to elucidate mechanisms of drug resistance in *Candida* fungi.

### Data availability

The sequences of partially phased primary contigs have been deposited in GenBank within BioProject PRJNA664297.
